# A review of the reliability and validity of the Edmonton Symptom Assessment System

**DOI:** 10.3747/co.v16i1.261

**Published:** 2009-01

**Authors:** L.A. Richardson, G.W. Jones

**Keywords:** Symptom screening, esas, review, reliability, validity

## Abstract

**Background:**

Systematic symptom reporting by patients and the use of questionnaires such as the Edmonton Symptom Assessment System (esas) have potential to improve clinical encounters and patient satisfaction. We review findings from published studies of the esas to guide use of the system and to focus research.

**Methods:**

A systematic search for articles from 1991 through 2007 found thirty-nine peer-reviewed papers from 25 different institutions, thirty-three of which focused on patients with cancer. Observations, data, and statistics were collated according to relevance, reliability, validity, and responsiveness.

**Results:**

Findings apply predominantly to symptomatic palliative patients with advanced cancer who were no longer receiving active oncologic therapies. Uncertainty about summarizing findings arises from frequent modification of the esas (altered items, scales, and time periods). Overall, reliability is established for daily administration. Scores are skewed, with a floor effect, but the relative order of symptoms by mean scores is similar across studies. Emotional symptoms are poorly captured by the depression and anxiety items. An equally weighted summation of scores may estimate a construct of “physical symptom distress,” which in turn is related to performance status, palliative goals, quality of life, and well-being.

**Conclusions:**

The esas is reliable, but it has restricted validity, and its use requires a sound clinical process to help interpret scores and to give them an appropriate level of attention. Research priorities are to further develop the esas for assessing a greater number of important physical symptoms (and to target “physical symptom distress”), and to develop a similar instrument for emotional symptoms.

## 1. INTRODUCTION

Patients with cancer experience many physical and emotional symptoms. When these patients self-report their symptoms, the prevalence and severity data for the symptoms tend to vary significantly from those identified by health care providers and from the data recorded in charts and on research forms 1. Patients may be better able to identify and assess symptoms that have a larger subjective component—for example, pain or fatigue. Discrepancies in clinical priorities and symptom subjectivity may explain the dissatisfaction with physical care registered by 20% of cancer patients in Ontario, and the dissatisfaction with emotional care registered by 43% (2004–2006) 2. Systematic symptom assessment tools completed by patients just before ambulatory and in-hospital clinical visits have a potential to shape encounters and may improve rapport, efficiency, informed consent, administered care, and patient compliance, and likewise, satisfaction with care.

A number of screening instruments for symptoms have been introduced. These range from single-focus tools to instruments that target multiple domains, and from checklists to multiple-item indices with complex scoring methods. For instance, some measures aggregate items into a summary score that may be linked to a concept such as distress or quality of life (qol). For patients with cancer, only a limited set of instruments are available for possible clinical use 3. These include the Distress Thermometer (dt)^4^, the Memorial Symptom Assessment Scale (msas)^5^, and the Edmonton Symptom Assessment System (esas) 6. Tools more prevalent in research contexts include the European Organization for Research and Treatment of Cancer (eortc) tools 7 and the Functional Assessment of Cancer Therapy (fact) tools^8^, although these may also be used in clinical practice.

The esas was first described in 1991 6 as a way to audit symptoms for patients on palliative care wards daily. This single-page screening tool has been praised for its brevity and ease of use 9, and is frequently described as practical, reliable, and valid 10–18. Cancer Care Ontario recently implemented the esas in cancer clinics across the province, intending administration at every patient visit 19. Some clinics in Ontario already have experience with the esas, but others have yet to use it as a process of care. Implementation requires interpreting the esas and having acceptable responses to the information it provides.

The purpose of the present review is to summarize what is known about the esas; to understand its reliability, validity, and interpretive schemes; to guide its use by patients and health care providers; and to prioritize research.

## 2. METHODS

In January 2008, we conducted a systematic search of the medline and pstycinfo electronic databases for published papers containing “Edmonton Symptom Assessment” in the title or abstract. A hand search was conducted for articles in personal files and for citations in papers, textbooks, and reference books. More than 130 relevant articles and abstracts were identified, of which thirty-nine were primary studies published as complete peer-reviewed papers, representing 25 different institutions. Of the thirty-nine papers, thirteen were from Canada 6,11,20–30; eight were from the United States 12–14,18,31–34; eight were from Scandinavia (Sweden 15,35–37, Denmark 38–39, Norway 40, Finland 16); two were from Western Europe (Netherlands 41, United Kingdom 42); five were from southern Europe (Switzerland 43, Italy 17,44–46); two were from Australia 47,48; and one was from Asia 49.

Data on patient demographics, cancer types, clinical contexts (for example, in-hospital, advanced disease), manner of use or modification of the esas, observed reliability and validity, and other observations relevant to use of the instrument were systematically extracted from each paper.

## 3. RESULTS

### 3.1 Population Characteristics

Of the thirty-nine papers, thirty-three represented studies conducted predominantly or exclusively in patients with cancer. The remaining six papers included other populations (patients with aids 13, renal disease 24, benign disease 29, and cardiorespiratory diseases 18,22,34). Sample sizes in the thirty-three oncologic studies ranged from 32 to 1296 subjects, for a total in excess of 5000 patients. However, eleven of the thirty-three studies reported some data for the same patients: Sunnybrook Cancer Centre in Canada 25–28, Bispebjerg Hospital in Denmark 38–39, Linkoping Hospital in Sweden 15,35,36, and Yale University 18,34. For the patients described in the thirty-three papers, median age ranged from 53 to 74 years, and men were in a slight majority overall. Most studies used the esas in its English or northern European language versions, and most of the study subjects were white.

Of the thirty-three oncologic studies, twenty-nine were conducted in palliative populations with advanced cancer, and more than one half of these studies administered the esas exclusively to hospitalized patients for acute admission, for time-limited and long-term admissions, when in intensive care, and with symptom acuity or with stable disease. The most frequent diagnoses were lung and gastrointestinal malignancies, although many other cancer types were represented. The overall distribution was perhaps typical of a palliative population. In the non-hospital but palliative context, most subjects were at home ^17,18,22,25–^30,32,34,36,40,47 or at a hospice 48. Overall, the “extreme” condition of the subjects is reflected in the descriptions provided in the papers themselves: disease was “advanced,” patients were “terminally ill,” symptoms were “moderate” to “severe” (justifying hospitalization), performance status was intermediate-to-low [median Karnofsky performance status (kps): 40–70], and reduced cognitive functioning was present in some patients. Longitudinal studies reported that one half of all patients dropped out from questionnaire completion within weeks during follow-up. Where reported, median survival was less than 3 months.

Of the twenty-nine studies in palliation, five included patients receiving active cancer therapies apart from supportive and palliative care: four targeted patients managed with radiotherapy (of which 75% were outpatients 25–28), and one studied survival with palliative chemotherapy 44. Finally, the remaining four of the thirty-three oncologic studies focused on less-palliative situations: one study looked at participants of three randomized trials, including 48 survivors of cancer 14; one included a “general cancer population” of 240 patients being managed with curative intent 31; one included patients having lymphoma or leukemia (although, on average, more than 2 years after initial diagnosis and in hospital) 33; and one examined patients between 6 and 36 months after initial diagnosis 37.

### 3.2 Face and Content Validity

Despite relative uniformity of the clinical contexts in which the esas was administered, its format and the symptoms that were included varied. Authors described these as “modifications” that included changed items, added items, and changes in descriptors or scales.

Typically, symptom scales were presented horizontally—a presentation that may make the esas seem simple and consistent to patients 47. However, the orientation of visual analog scales (horizontal vs. vertical) may have a critical influence on measurement. This factor requires further investigation with the esas 50. Noticeably distinct from the original esas was replacement of a visual analog scale (0–100 mm) with categorical numbered scales (0–10). One study further reduced scales to 5 categories 12, and others reduced them to 4 categories 18,34. No comparisons of these versions of scales have been published, although some authors have claimed that visual analog and numeric scales elicit similar scoring 51. Numeric scales may be more acceptable to patients, more efficient to score 31,42,48, and applicable from bedside care to telephone follow-up 25.

Another noticeable modification to the esas was the period over which symptoms were to be scored. This period has varied from as short as “at present” or within the past few hours 51, up to several days 48 or weeks 25,37. Most studies administered the esas daily, without explicitly defining a period of assessment. The importance of being specific is clear for the “severe pain” item, where “severe pain” is understood by patients to mean “worst pain” rather than some other statistic or estimate 31, and the influence of time period on all items remains a concern.

The number of symptoms in the esas has varied from 8 items (in the original version) to more than 11—typically with 1 “open” scale for an additional rater-selected symptom. The most recent version of the esas includes 9 symptoms—(in order) pain, tiredness, nausea, depression, anxiety, drowsiness, appetite, well) being, and shortness of breath (sob 52. A score of 10 is described as the “worst possible” symptom. This recent esas version includes 7 of the 12 most common symptoms identified in large surveys 9,53,54. These symptoms may be common, but they need not be the most distressing symptoms 35—except for pain, which has been established to be important for palliative patients 55. Across all thirty-three oncologic studies reviewed, only 3 symptoms were included in almost every version of the esas: pain, depression, and anxiety. All other symptoms have been replaced with alternatives, including gastrointestinal problems such as constipation 13,16, and sleep-related problems such as insomnia or difficulty sleeping 12,16,32,41. When new items were added to the esas 18,29,34, investigators sometimes found those items to be more clinically appropriate.

Discussion of content brings into focus a question of whether the esas domains exhibit “balance.” In all versions, physical symptoms outnumber items that may represent psycho-emotional symptoms. Patients are likely to perceive the focus to be physical symptoms. However, these symptoms (typically, reduced appetite, nausea, drowsiness, tiredness, pain, and sob) may sometimes be manifestations of psychological disorders or components of psychiatric diagnoses. Further, the terms “anxiety” and “depression” may be relatively salient because of their connotations—that is, are these folk and non-stigmatizing language, or are they psychiatric labels? Studies of the esas reveal significant changes in depression scores within days 11,13, which suggests that patients interpret “depression” as a dynamic process, a feeling, or an emotion 11—although in a few studies, the change may simply be a regression-to-the-mean bias arising from the subgrouping of patients by initial scores. Some investigators have used “feelings of depression” as the descriptor 18,34.

Finally, qol and well-being may not be legitimate symptoms or items for the esas^15^,^31^. First, they may not transfer accurately across cultures and languages 17. Second, well-being may be understood to be a more psychological term—an outcome of care and not a symptom. It was originally considered to be an internal summary for all other items 51, which raises a second aspect of “balance” between measuring negative symptoms and deficits, and measuring positive strengths and attributes. The focus of the esas is the former, but a strict opposition or correspondence between negative and positive domains is not a requirement. Both may be needed for clinical management.

### 3.3 Theoretical Validity

The literature provides no theoretical justification for the esas and its content. An argument may be made that a theory or an overall meaning to the esas is not necessary, because the goal may simply be practical: to identify a few active symptoms using a consistent listing and scoring system across patients.

### 3.4 Score Distributions

Summary baseline esas scores were provided in more than half of the studies reviewed, but one third of the thirty-nine papers did not provide mean scores or distributions by individual items. Baseline average scores ranged from a low of 6 to a high of 75 (out of 100) for all symptoms, but nausea was consistently the lowest-scoring item (range: 6–28), and activity level (range: 49–75), fatigue (range: 44–67), appetite (range: 44–61), and qol [47 (only one study)] were the highest. In general, mean scores were typically less than 50 out of 100, with distributions exhibiting significant “floor” effects 14,16,17,27,31. These distributions were not statistically “normal” (being skewed, or having multiple modes) 16. Means may vary with socio-ethnicity 33. Mean scores this low would not be expected in palliative studies, in which investigators describe symptoms as moderate-to-severe (although the most severe symptoms might not be captured with the esas). Possibly, some patients interpreted the 11-point scales as having fewer categories, were unable to distinguish adjacent categories, or failed to understand the scale as linear and strictly interval. Low mean scores in the esas reflected scores of exactly 0 (that is, no symptom) given by 40%–57% of palliative patients on some items 16,17,31, although fewer than 2% of patients scored 0 on every symptom 16,27,35. Reinforcing the floor effect, investigators differed with regard to the values that they took to mean symptom absence (alternatively, prevalence), because some used 0 to mean that a symptom was absent, and others used a range of values (for example, 0–2 meant either no symptom or not a clinically significant symptom) 56.

### 3.5 Reliability

Studies in which multiple instruments were co-administered with the esas looked at a very short time of administration, and they explored the effects of various formats, time periods, scales, wordings, and anchors when asking similar questions about a symptom. This type of reliability is often labelled “concurrent validity,” because it demonstrates a reproducible score despite variation in presentation.

Correlations between individual esas items and scores elicited by alternative tools are fairly good, particularly for physical symptoms. Chang *et al.* 31 showed that the esas symptoms correlating most strongly with msas symptoms were sob (0.85), appetite (0.75), nausea (0.62), nervous (0.45), and depression (0.44). Similarly, Moro *et al.* 17 showed some esas symptoms correlating strongly with items in the Symptom Distress Scale (sds) 57: nausea (0.88), sob (0.84), pain (0.80), appetite (0.74), and depression (0.64). Moderate weighted kappa values were reported by Philip *et al.* 47 for agreement with the Rotterdam Symptom Checklist 58, including sob (0.58), anxiety (0.48), appetite (0.46), and depression (0.45). Correlation of the Brief Pain Inventory 59 and pain was 0.61 with the esas item most associated with “worst pain.”

All of the foregoing findings suggest that presentation and embedding questions inside other instruments change scores, even for physical symptoms. Also, in the context of a short time span, when patients were prompted to reconsider their esas scores by physicians, the inter-rater weighted kappa analysis showed strongest agreement for well-being (0.78), anxiety (0.72), and depression (0.71). Overall, however, patients were prone to revise scores up and down equally 32. Patients may be influenced by the immediate context (that is, the people present) or by a challenge, but this situation could be an aspect of reliability or a method to improve validity.

Reliability by test–retest with the esas and within 1 day is generally high, exceeding 0.8 6,11,12,31,42–43, with the exception of one report of 0.35–0.72 21. Collectively, studies have concluded that the esas need not be administered more than once daily for most palliative patients, even when in hospital. Repeated measurements that are a full day apart give contradictory results 17,21, representing lower reliability or a responsiveness to change. (Because of the dynamic nature of symptoms in very palliative contexts, we consider repeated measures over longer periods of time, from 3 to 30 or more days, in the section on responsiveness.)

Proxy ratings support restricting the esas to eliciting symptoms directly from patients. Detailed studies by Nekolaichuk 20,21 demonstrated that physicians assign scores that are lower and nurses assign scores that are similar to those of the patient. Lower correlations were apparent for subjective items of anxiety, depression, and well-being. Correlation coefficients for physician-or-patient ratings were weakest for activity (0.33), depression (0.45), and well-being (0.44) 20; for nurse-or-patient ratings, coefficients were weaker overall, with 0.35 for activity, 0.37 for depression, and 0.23 for well-being. Interestingly, proxy scores did not converge with patient self-scores over 2 weeks of follow-up.

Another study 43 reported that physician and nurse assessments were similar, but that both differed from patient ratings. Interestingly, providers underestimated physical symptoms and overestimated well-being, anxiety, and depression. Physician-or-patient ratings had relatively weak Pearson coefficients for drowsiness (0.33), well-being (0.44), and depression (0.49), and were non-significant for nausea and anxiety. Likewise, coefficients for nurse-or-patient associations were weak for sob, drowsiness, and depression (0.32, 0.43, and 0.43 respectively).

Finally, an estimate using generalizability theory 20 suggested that 3 raters on 1 occasion or 2 raters over 2 occasions within 1 day are required to achieve acceptable reliability. This reliability hypothesis would apply only if patient self-reporting were not the purpose of the esas.

### 3.6 Factor Analyses, Internal Consistency, Summated Scoring, and Construct Validity

Associations between pairs of symptoms can imply “structure”. Chow *et al.* 28 found significant Spearman correlation coefficients between all 9 symptoms (each *p* < 0.0001, coefficients ranging from 0.15 to 0.68). Similarly, Heedman and Strang 15 found correlations between anxiety and depression (0.76) and between well-being and qol (0.64). However, Teunissen *et al.* 41 found no correlation between emotional and physical items.

At a larger scale than pair-wise associations, fatigue, tiredness, sleep difficulties, and drowsiness might be predicted to form one statistical factor; anxiety and depression, another; and appetite and nausea, a third. Three factors were uncovered at baseline for patients with bone metastases who completed the esas before radiation therapy 28: fatigue, pain, drowsiness, and well-being; anxiety and depression; and nausea, appetite, and sob. Each factor had internal consistency (Cronbach alpha values ranging from 0.68 to 0.80). During follow-up after radiation, the factor structure changed, making it impossible to generalize a factor structure for the esas from this single study 28. Studies in a mixed population of patients, including some with cancer 18,34, confirmed that an emotional factor was distinct from a physical factor, but otherwise, that any structure was unclear and not aligned with clinical experience. More research is required to determine whether a factor structure exists and in which specific clinical contexts it might apply.

Under an assumption that the esas has no factors, the “global” internal consistency has been studied by calculating an overall Cronbach alpha, resulting in values of 0.79 31, 0.80 29, and 0.93 48. These results suggest that items are interrelated and that a combination represents a latent construct. However, it has been noted that, if the esas estimates qol (for instance), then it is not necessary that a patient suffer from all symptoms to have a poor qol, and that correlation-based methods will be inappropriate for scale validation 17. Even so, many investigators have followed the original suggestion 6,51 to summate item scores with equal weighting into an index [Symptom Distress Score (esas-sds)], giving values between 0 and 100 for 10 symptoms. It was originally suggested that the summary score may represent the construct of “symptom distress,” leading to the esas-sds name 6. (Note that this terminology should not to be confused with a definition of distress provided by the National Comprehensive Cancer Network, which definitely has an emotional component 60, nor with another instrument, the 14-item Symptom Distress Scale 17,57).

Under the assumption of one latent construct, studies of “construct validity” have been undertaken. The meaning of construct validity varies, and for the present review, we have taken it as an indication of whether the esas estimates a physiologic or psychological construct that is difficult to measure directly. No underlying theory or “gold standard” instrument provides a comparison 47 (that is, criterion validity), but the evidence for an esas construct (“physical symptom distress”) is set out as follows:

- In 9 healthy subjects 37, esas scores were lower than they were for patients with cancer. Another study compared symptoms for patients with cardiorespiratory illnesses and cancer, and demonstrated potentially comparable burdens of disease 34—perhaps more with respiratory disease than with heart disease.
- The esas-sds distributions for hospitalized palliative patients were greater than those for palliative outpatients, with values ranging from 35 to 45 (out of 100). The strongest evidence is provided by the mean esas-sds and item scores, which differed by location of care for patients managed at the same institution (in several concurrent nonrandomized cohort studies) 17,27,31,39. Data from a single cohort series of patients during curative treatment had very low esas sds scores 31.
- The esas-sds correlated with the msas global distress index (0.73, *p* < 0.0001) and more with the physical symptom subscales of the fact and the msas 5 than with the psychological subscales 31. Symptom prevalence was greater with the esas than with the msas and the fact, but the esas-sds had a strong correlation with the msas physical subscale (0.74), followed by those with the global distress index (0.73) and the total msas score (0.72). A weaker yet statistically significant correlation was observed between the esas-sds and the msas psychological subscale (0.56).
- The Symptom Distress Score 57 was correlated with the esas-sds (0.77), but physical items correlated better than psychological items did 17.
- Studying a renal disease population rather than a cancer population, Davison *et al.* 24 demonstrated that esas scores varied with the burden and the effects of disease, but not with biochemical markers. The esas-sds correlated with qol (coefficient of 0.69).

All data regarding possible structure and internal consistency of the esas suggest that the esas-sds may estimate a latent construct of “physical symptom distress.” Whether such a construct is merely physiologic or whether it has a significant emotional component is not established.

### 3.7 Convergent and Divergent Validity

The esas-sds has been compared and contrasted with summary indices from other instruments that represent other constructs:

- ***Performance Status*** The esas-sds was associated in several studies with kps 15,27,31, but not with the Eastern Cooperative Oncology Group (ecog) scale ^29.^
- ***Emotional Disorders*** First, the esas and the Hospital Anxiety and Depression Scale (hads) 61 have been repeatedly compared. Studies have found only a modest association or no association between them 14–15,35,37,41. The individual items of “anxious” and “depressed” do not map well to results with hads, and no valid cut-offs have been established for screening with those two esas items 14,41. In contrast, (although in a non-cancer population with renal disease), Davison *et al.* 24 determined that the esas-sds correlated more strongly with mental (–0.62) rather than with physical (0.54) health, as measured using the Kidney Dialysis Quality of Life Short Form 62. The physicality of the esas may therefore not be a general phenomenon outside oncology. Second, two recent abstracts (no full publication as yet) reported only moderate correlation (0.45) between the esas-sds and the dt in patients with cancer 63, and only moderate correlations for items such as qol (0.48), nervousness (0.45), and depression (0.44) 64. Notably, dt is intended to estimate emotionally-related distress 60.
- ***Goals of Palliative Care*** Bruera 51 demonstrated that the esas-sds was associated with the 17-item ) Support Team Assessment Schedule (stas 65,66. The stas assesses insight, communication, planning, and aid. It is a general measure of aspects of caring and the organization of care, and it has only 4 items regarding physical and emotional symptoms. The stas and the esas-sds had a correlation of 0.8 (*p* < 0.0001).
- ***Quality of Life*** The esas-sds was associated with qol in several studies 15,29,35,48. Tierney *et al.* 48 compared the esas with the McGill QOL Questionnaire 67. The esas-sds correlated with the overall McGill scale (*p* < 0.0001), with physical well-being, and with the psychological scale (*p* < 0.0001). Physical symptoms of the esas did not correlate with the McGill physical domain. A number of investigators co-administered other qol instruments (eortc-Q30 38–40 and eortc qlq-pan-26 40 and 15D qol 16), but they did not provide statistics comparing these instruments directly with the esas. Others who directly compared the eortc Q30 and qlq-pan-26 with the esas found that patients preferred completing those instruments rather than completing the esas 29, but no reason was provided.
- ***Well-being*** Well-being was typically included in the esas, and it is strongly associated with the overall esas-sds. This finding was interpreted to mean that the response to this item may be an overall outcome of the tool 6,15, supported by the finding that well-being was associated with the physical symptom scores. As an item, its location within the esas (that is, surrounded by physical symptoms) means that the item of well-being is not a true test of construct validity or of convergence–divergence. However, Chang *et al.* 31 compared the esas-sds with the fact general scale and found the esas-sds to be most correlated with fact physical well-being (–0.75), followed by qol (–0.69), functional well-being (–0.63), and finally emotional well-being (–0.52).
- ***Patient Satisfaction*** It was impossible to demonstrate a statistical association between esas scores, which exhibited floor effects, and patient satisfaction, which exhibited ceiling effects 48.

In summary, the esas-sds is moderately associated with performance status, palliative care goals, qol, and well-being, but it is minimally associated with emotional symptoms and may not be associated with patient satisfaction.

### 3.8 Other Statistical Associations

Another aspect of validity is whether the esas is associated in predicable patterns with a diversity of variables and clinical events.

First, Bradley *et al.* 27 noted that younger patients reported greater anxiety scores, that patients with greater weight loss reported higher symptom scores, and that pain score was associated with opiate use.

Second, the probability of documentation of symptoms in the medical chart was greater when patients reported higher esas scores, suggesting that severity of symptoms is associated with greater scores 38. However, although patients in another study scored the presence of symptoms, fewer of them were on medications appropriate for those symptoms 30. Some investigators interpreted this as clinical failure, variation in practice 36, or justification for palliative care consultation 44. Such interpretations assume, rather than establish, the validity of the esas.

Third, several studies investigated whether the esas-sds or separate esas items associate with disposition from consultation and with overall survival. These analyses are complicated by the possibility that esas scores may have helped caregivers decide subsequent clinical disposition (for example, into tertiary care) 22. Empirically, greater esas scores and reduced survival are associated in several studies 15,22,49, but not in others 44–45. Patients with greater esas scores may want fewer active interventions to prolong life. Further, whether the esas adds unique information relative to kps 27,31 is not clear, although this association of the esas and kps was not evident in some studies 17,29,49. Specific items on the esas are known to independently predict survival (physical symptoms such as reduced appetite and greater dyspnea, for example) 15,44. Although the esas assesses those symptoms and so has the potential to be associated with overall survival, the primary purpose of the esas is to manage symptoms or to audit practice, regardless of expected survival.

### 3.9 Responsiveness

The esas has been repeatedly administered to the same sets of patients at between 2 and 30 or more days of follow-up. Longitudinal scores can be found in fifteen papers 6,11,15,17,23,25–29,33,40,42,45,46. Given that some patients were admitted for great acuity of symptoms and for immediate care and benefit, and that others experienced very short survival times with increasing difficulties in completing the esas, such studies may be understood as tests of positive and negative responsiveness. However, other interpretations are certainly possible: for example, change in patient priorities, reinterpretation of symptoms and anchors, and sometimes simply a bias of regression to the mean as a result of the method of statistical analysis.

Many studies claimed that interventions were “successful” in reducing esas item scores and the overall esas-sds, but not all studies found mean scores to decline as predicted. Most studies did not tally or measure interventions, and they provided no data for associations between interventions and changes in scores 6,11,13,15,17,31,42. Heedman and Strang 36 showed no association between pain level and changes in pain medications, but others showed that some item scores responded to relevant interventions. For example, a few selected case studies presented by Bruera 23,51 nicely demonstrated the graphical use of esas item scores over time to monitor symptoms and to document effective care of individual patients. For populations of patients, anxiety and dyspnea declined in response to paracentesis for ascites, and both were related to self-reported overall improvement 29. Detailed studies by Chow 25,26,28 provide telephone-based follow-up scores and sequential factor analyses for up to 12 weeks of follow-up subsequent to radiotherapy. These follow-ups demonstrated changes in scores and statistical factors, although the subject drop-out rate was high.

Missing data make conclusions fragile and potentially biased, but in comparing patients who responded to radiation with patients who did not, the baseline esas symptoms did not predict response to radiation, although scores diverged in the two groups during follow-up 28. For art therapy 33, the esas-sds mean value declined, but fewer than half of the patients approached to participate did so, and participants were favourably disposed to art therapy at baseline and may have overestimated the benefit. Therefore, from these studies, the overall impression is that the responsiveness of the esas remains questionable.

How frequently should the esas be administered to patients to measure change and interpret symptoms in follow-up 10?

Standard frequencies are not established for patients with various types of cancer, specific symptoms, and care-delivery contexts. Only in palliative contexts, and especially in hospitals, is daily esas administration reasonable, but even in that context, concern was raised regarding the benefits of finely tracking symptoms when these patients are deteriorating near the end of life 26,40,42. For example, patients stop completing the esas in advance of death because of either inability or lack of perceived benefit. Labori *et al.* 40 reported increased symptom intensity in the last 8 weeks before death, citing the natural elements of the terminal phase of disease that inevitably worsen. Dudgeon *et al.* 11 showed that overall esas scores improved within 2 days, but subsequently deteriorated. Unfortunately, no studies have been performed in patients receiving adjuvant curative therapies to establish a frequency of administration.

## 4. DISCUSSION

The esas is a relatively new instrument. A number of investigators have explicitly claimed that it has established reliability and validity 11–17, citing several papers 6,31,47. However, Bruera *et al.* 6 clearly identified the first paper on the esas as being only a “description of initial experience” and provided a limited statistical analysis that focused on the summary esas-sds and the value of the esas as an overall tool for auditing care for a population of patients. Hearn *et al.* 3 reviewed 41 symptom-screening instruments through 1997, including the esas, and concluded that, of the 12 most relevant instruments, not one was comprehensive or ideal. Further criteria for appraisal were listed by Sloan *et al.* 68, and many reports concerning the esas have been published since 1997.

Our review compiled a total of thirty-nine studies published through 2007. Of these, thirty-three were conducted predominantly or exclusively in the context of cancer. TABLE I provides our conclusions, and we use the remainder of this discussion to explore implications for the use of the esas, its further development, and innovation to address emotional symptoms.

### 4.1 Using the ESAS

One possible perspective on the esas, without making further modifications, is to use the tool as a limited but systematic extension of the conventional, indeed the venerable, “review of systems.” For this task, a “standard version of esas” would ensure that earlier research findings apply. Evidence suggests that proxy assessments are not substitutes for patient self-scoring of symptoms, and so the esas should be administered antecedent to clinical visits. The esas is brief, practical, rapid, and visual, and a numeric horizontal scale (0–10) may be the best format. The time period to which the questions apply should be explicit. Score reliability is established for daily administration in the context of palliation, in particular for certain language groupings (English, for instance) and socio-ethnic backgrounds (for example, white) with advanced lung and gastrointestinal malignancies, many of whom are hospitalized. Discordance is noted between clinical impression (for example, severe symptoms) and actual patient scores (for example, a significant floor effect and low means in the score distributions); between in-chart documentation and esas scores; between the timing and intensity of interventions and any change in esas scores; and between esas scores on anxiety and depression and scores from screening tools such as hads. Therefore, to determine the patient’s meaning for any esas score (for example, whether the scale is interval or merely ordinal, whether drift or resetting of anchors occurs, and whether symptoms of greater concern are not included in the esas) requires dialogue to turn possibly reliable and valid information into shared knowledge. Consequently, this tool has to be embedded within a strong clinical context such that esas values can be dialogically validated and translated into actions.

A patient’s perceptions, interpretations, desires, and expectations must be understood. First, the plain meaning of all item scores has not been fully established—for example, nausea could mean the most severe recent nausea—although this is more a matter of refinement of the instrument. Second, the esas may empower patients only if it is relevant to them and if the clinical team does it justice by paying attention to it. Third, the meaning of an equally-weighted summary index (esas-sds) may be consistent with a latent construct of physical symptom distress (negative dimension) or with physical well-being (positive dimension). Besides responding to the score for each individual item in isolation, understanding and tracking of the summary index of the esas-sds seems appropriate.

We recommend using a standardized esas with palliative patients, especially with those who are hospitalized and who remain capable of completing the instrument. The optimal way to embed the esas into clinical processes and structures is still to be determined. Symptoms may then be linked with practitioners in respective clinical disciplines (for example, nutrition, psychology). The purposes for which the esas is best suited are the management of physical symptom assessment, clinical audit, and program development in supportive care (for example, by gathering information).

### 4.2 Further Developing ESAS to Address Physical Distress

We suggest further exploration of the role of the anchors and descriptors of the esas items to improve clarity (for example, time period, with or without a consideration of the effect of interventions) and to redistribute scores (for example, “upwards”) for statistical and interpretative purposes. Also, a test should be made of whether a simpler scale (fewer categories than the 11 sometimes used, or a 100-mm line) is better than revising anchors and time periods.

One practical approach to the esas is to focus on managing the items with the most extreme values (whatever their absolute scores), and another is to understand the larger set of symptoms, even when some are only “mild” in severity. The ability to detect prevalence for these purposes requires appropriate scaling, anchors, and descriptors, possibly with labels attached to numerically intermediate values.

We recommend that the number of physical symptoms be increased from 7 to at least 10, with items such as constipation and dry mouth being possible additions with wide relevance. In addition to large systematic surveys of symptoms, qualitative research (that is, focus groups) may be required to identify important items. Expanding the list of symptoms would reduce the need for investigators to constantly modify the esas or to supplement it with interviews for additional symptoms. However, the physical symptoms privileged by their inclusion in the esas should not be redundant (that is, from among the same symptom clusters or statistical factors), so that the esas can remain brief, efficient, and simple. The esas should target the symptoms with greatest meaning or clinical implication—that is, those that cause important physical symptom distress reliably representing clustered symptoms that can be managed or treated or that have prognostic significance. To increase internal consistency (or factorization) and to strengthen the esas-sds in its estimation of “physical symptom distress,” we recommend removing items such as qol, well-being, anxiety, and depression from the esas and replacing them with more physically-oriented items. Conceivably, items could also be added to assess the effect of physical symptoms on other dimensions (for example, cognition, spiritual suffering, and the emotions representing disruptive physical symptoms), and to determine physical strengths (for example, exercise capacity or tolerance) that may help in management.

Although physical symptoms may interact and complicate management, a “first approximation” to managing core physical symptoms is to assume that they are entirely physical and can be effectively managed in isolation. This practical approach is an extension of the current model of supportive care. Cut-off values to categorize severity (mild, moderate, severe) have not been validated for the esas, and so sorting patients for various interventions by ranges of scores is problematic. There are probably more esas values (11) than there are clinical responses. Although physical symptoms and processes are the focus, interventions could include psychosocial and behavioural actions—for example, managing fatigue and sleep disturbances.

To build positively on the existing success of the esas, a revised esas is a research priority. To establish reliability and validity, a cumulative research program will be required, particularly in the contexts of newly diagnosed patients and ambulatory care.

### 4.3 Addressing Emotional Symptoms

We conclude that the emotional components in the esas are underdeveloped and require extensive modification. Patients recently diagnosed with cancer and those undergoing treatment for cancer often suffer high levels of emotional symptoms, with many patients further developing clinical disorders of anxiety, depression, and post-traumatic distress 69. Many experience difficulty coping that extends into survivorship: one quarter of all cancer-free survivors suffer from psychiatric disorders 69. A patient with cancer will not experience isolated symptoms with individual causes, but will experience a complex combination of interacting physical and emotional symptoms regardless of causation, which complicates management. When only a physiologic perspective is used, considerable difficulty arises in interpreting scores for physical symptoms. And even if the meaning of the scores are the same across patients, those patients may not report the same scores when experiencing the same level of symptoms. Some patients may not want to divulge symptoms that can jeopardize cancer treatment (for example, depression); others may want to avoid symptoms, being unable to face the mortality that such symptoms imply (emotional “repressors”) 70. For these reasons, each item’s score or range of scores may have various meanings and correspondingly various implications for care. This understanding reinforces the established observation that no clear cut-offs for screening have been determined for the esas scales. The rich information that an 11-point esas scale for physical symptoms provides to the clinician would be lost by simplistic reduction to errant categories of mild, moderate, and severe, and by ignorance of the associated emotions and meaning.

To address emotional symptoms, we recommend innovation through the development of a separate tool, similar to the esas, that addresses 10 or more emotional symptoms, that uses nonpsychiatric labels, and that encompasses symptoms representing more disorders than just anxiety and depression. This new instrument would inject the esas into a theoretical framework in which physical and emotional symptoms associate for reasons of causality and interaction and for which interpretation of item values requires, at a minimum, an understanding of both physiology and psycho-emotional processes. Whether an existing “emotional” tool may be sufficient for this task is unknown. The hope is that concurrently administering a complementary pair of esas-physical and esas-emotional questionnaires could provide a more nuanced and useful interpretation for front-line staff.

The interjection of a new technology such as the esas presents methodology, resource, interpretative, scope-of-practice 71, and ethical-medico-legal challenges 72. We consider the esas to be a step in the right direction, but research regarding validity of the esas outside a palliative context is limited. Further elaboration of the esas with the goal of measuring more physical symptoms and related “distress,” and of measuring emotional symptoms are research priorities.

## Figures and Tables

**TABLE I:** Conclusions regarding the Edmonton Symptom Assessment System (esas)

Domain	Conclusion or conclusions
Population characteristics	Findings on reliability and validity are limited to the palliative cancer population receiving supportive and palliative care only, especially hospitalized patients. Data in other clinical contexts and for most languages are insufficient.
Face and content validity	Too many versions of the ESAS exist, introducing an unquantifiable concern when aggregating findings about reliability and validity across studies. The “standard” ESAS is a short patient self- report that identifies some of the more common physical symptoms.
Theoretical validity	The ESAS was not psychometrically derived within a theoretical framework. It may not be feasible to “retrofit” a larger meaning to it from empiric findings. Lack of a theory makes the task of identifying constructs or predicting statistical associations difficult.
Score distributions	Skewed distributions improve reliability because serial responses are similar 23, but they reduce the statistical capacity to validate the ESAS through tests of association and to establish responsiveness 16,48. Very low symptom scores are of concern in a nonpalliative setting, in which symptom absence ranges from 22% to 81% 31. Scales and anchors might be adjusted to try to improve score distribution.
Reliability	Concurrent instruments give moderate-to-good correlations for physical symptoms, but lower correlations for psychological–psychiatric items. The ESAS may be administered once daily in most palliative contexts.
Structure of the ESAS	By Cronbach alpha and factor analyses, the ESAS may have an internal structure, with unknown nature, extent, and meaning. A summary score [ESAS–SDS (Symptom Distress Scale)] may estimate “physical symptom distress.” Research into whether distress is the appropriate construct (for example, to determine effect on emotions and higher functions such as cognition and spirituality) is required.
Convergent and divergent validity	More data are needed. The ESAS–SDS targets physical symptoms and is associated with performance status, quality of life, physical well-being, and palliative care goals. The ESAS is less associated with psychological and psychiatric domains. Items of anxiety and depression have no established cut- offs for emotional–psychological screening.
Expected statistical associations	This area has been poorly studied.
Responsiveness	Responsiveness is a function of reliability and validity. Scores on the ESAS vary inconsistently with interventions. The frequency of completing the ESAS to measure change is unknown.

## References

[1] Davidson SE, Trotti A, Ataman OU (2007). Improving the capture of adverse event data in clinical trials: the role of the International Atomic Energy Agency. Int J Radiat Oncol Biol Phys.

[2] Cancer Care Ontario (CCO) and Action Cancer Ontario (2007). Patient satisfaction with outpatient cancer care, Toronto Central lhin [Web page].

[3] Hearn J, Higginson IJ (1997). Outcome measures in palliative care for advanced cancer patients: a review. J Public Health Med.

[4] Roth AJ, Kornblith AB, Batel–Copel L, Peabody E, Scher HI, Holland JC (1998). Rapid screening for psychologic distress in men with prostate carcinoma: a pilot study. Cancer.

[5] Portenoy RK, Thaler HT, Kornblith AB (1994). The Memorial Symptom Assessment Scale: an instrument for the evaluation of symptom prevalence, characteristics and distress. Eur J Cancer.

[6] Bruera E, Kuehn N, Miller MJ, Selmser P, Macmillan K (1991). The Edmonton Symptom Assessment System (esas): a simple method for the assessment of palliative care patients. J Palliat Care.

[7] Aaronson NK, Ahmedzai S, Bergman B (1993). The European Organization for Research and Treatment of Cancer qlq-C30: a quality-of-life instrument for use in international clinical trials in oncology. J Natl Cancer Inst.

[8] Cella DF, Tulsky DS, Gray G (1993). The Functional Assessment of Cancer Therapy scale: development and validation of the general measure. J Clin Oncol.

[9] Strömgren AS, Groenvold M, Pedersen L, Olsen AK, Sjogren P (2002). Symptomatology of cancer patients in palliative care: content validation of self-assessment questionnaires against medical records. Eur J Cancer.

[10] Kirkova J, Davis MP, Walsh D (2006). Cancer symptom assessment instruments: a systematic review. J Clin Oncol.

[11] Dudgeon DJ, Harlos M, Clinch JJ (1999). The Edmonton Symptom Assessment Scale (esas) as an audit tool. J Palliat Care.

[12] Nelson JE, Meier DE, Oei EJ (2001). Self-reported symptom experience of critically ill cancer patients receiving intensive care. Crit Care Med.

[13] Brechtl JR, Murshed S, Homel P, Bookbinder M (2006). Monitoring symptoms in patients with advanced illness in long-term care: a pilot study. J Pain Symptom Manage.

[14] Vignaroli E, Pace EA, Willey J, Palmer JL, Zhang T, Bruera E (2006). The Edmonton Symptom Assessment System as a screening tool for depression and anxiety. J Palliat Med.

[15] Heedman PA, Strang P (2001). Symptom assessment in advanced palliative home care for cancer patients using the esas: clinical aspects. Anticancer Res.

[16] Salminen E, Clemens KE, Syrjänen K, Salmenoja H (2008). Needs of developing the skills of palliative care at the oncology ward: an audit of symptoms among 203 consecutive cancer patients in Finland. Support Care Cancer.

[17] Moro C, Brunelli C, Miccinesi G (2006). Edmonton Symptom Assessment Scale: Italian validation in two palliative care settings. Support Care Cancer.

[18] Walke LM, Byers AL, Gallo WT, Endrass J, Fried TR (2007). The association of symptoms with health outcomes in chronically ill adults. J Pain Symptom Manage.

[19] Cancer Care Ontario (CCO) and Action Cancer Ontario (2005). Provincial Palliative Care Integration Project: Resource Manual. Edmonton Symptom Assessment System (esas).

[20] Nekolaichuk CL, Bruera E, Spachynski K, MacEachern T, Hanson J, Maguire TO (1999). A comparison of patient and proxy symptom assessments in advanced cancer patients. Palliat Med.

[21] Nekolaichuk CL, Maguire TO, Suarez–Almazor M, Rogers WT, Bruera E (1999). Assessing the reliability of patient, nurse, and family caregiver symptom ratings in hospitalized advanced cancer patients. J Clin Oncol.

[22] Jenkins CA, Schulz M, Hanson J, Bruera E (2000). Demographic, symptom, and medication profiles of cancer patients seen by a palliative care consult team in a tertiary referral hospital. J Pain Symptom Manage.

[23] Bruera E, Michaud M, Vigano A, Neumann CM, Watanabe S, Hanson J (2001). Multidisciplinary symptom control clinic in a cancer center: a retrospective study. Support Care Cancer.

[24] Davison SN, Jhangri GS, Johnson JA (2006). Cross-sectional validity of a modified Edmonton symptom assessment system in dialysis patients: a simple assessment of symptom burden. Kidney Int.

[25] Chow E, Wong R, Connolly R (2001). Prospective assessment of symptom palliation for patients attending a rapid response radiotherapy program. Feasibility of telephone follow-up. J Pain Symptom Manage.

[26] Chow E, Davis L, Holden L, Tsao M, Danjoux C (2005). Prospective assessment of patient-rated symptoms following whole brain radiotherapy for brain metastases. J Pain Symptom Manage.

[27] Bradley N, Davis L, Chow E (2005). Symptom distress in patients attending an outpatient palliative radiotherapy clinic. J Pain Symptom Manage.

[28] Chow E, Fan G, Hadi S, Filipczak L (2007). Symptom clusters in cancer patients with bone metastases. Support Care Cancer.

[29] Easson AM, Bezjak A, Ross S, Wright JG (2007). The ability of existing questionnaires to measure symptom change after paracentesis for symptomatic ascites. Ann Surg Oncol.

[30] Riechelmann RP, Krzyzanowska MK, O’Carroll A, Zimmermann C (2007). Symptom and medication profiles among cancer patients attending a palliative care clinic. Support Care Cancer.

[31] Chang VT, Hwang SS, Feuerman M (2000). Validation of the Edmonton Symptom Assessment Scale. Cancer.

[32] Garyali A, Palmer JL, Yennurajalingam S, Zhang T, Pace EA, Bruera E (2006). Errors in symptom intensity self-assessment by patients receiving outpatient palliative care. J Palliat Med.

[33] Nainis N, Paice JA, Ratner J, Wirth JH, Lai J, Shott S (2006). Relieving symptoms in cancer: innovative use of art therapy. J Pain Symptom Manage.

[34] Walke LM, Gallo WT, Tinetti ME, Fried TR (2004). The burden of symptoms among community-dwelling older persons with advanced chronic disease. Arch Intern Med.

[35] Åstradsson E, Granath L, Heedman PA, Starkhammar H (2001). Cancer patients hospitalised for palliative reasons. Symptoms and needs presented at a university hospital. Support Care Cancer.

[36] Heedman PA, Strang P (2003). Pain and pain alleviation in hospital-based home care: demographic, biological and treatment factors. Support Care Cancer.

[37] Lindemalm C, Strang P, Lekander M (2005). Support group for cancer patients. Does it improve their physical and psychological well-being? A pilot study. Support Care Cancer.

[38] Strömgren AS, Groenvold M, Pedersen L, Olsen AK, Spile M, Sjøgren P (2001). Does the medical record cover the symptoms experienced by cancer patients receiving palliative care? A comparison of the record and patient self-rating. J Pain Symptom Manage.

[39] Strömgren AS, Goldschmidt D, Groenvold M (2002). Self-assessment in cancer patients referred to palliative care: a study of feasibility and symptom epidemiology. Cancer.

[40] Labori KJ, Hjermstad MJ, Wester T, Buanes T, Loge JH (2006). Symptom profiles and palliative care in advanced pancreatic cancer: a prospective study. Support Care Cancer.

[41] Teunissen SC, de Graeff A, Voest EE, de Haes JC (2007). Are anxiety and depressed mood related to physical symptom burden? A study in hospitalized advanced cancer patients. Palliat Med.

[42] Rees E, Hardy J, Ling J, Broadley K, A’Hern R (1998). The use of the Edmonton Symptom Assessment Scale (esas) within a palliative care unit in the U.K. Palliat Med.

[43] Pautex S, Berger A, Chatelain C, Herrmann F, Zulian GB (2003). Symptom assessment in elderly cancer patients receiving palliative care. Crit Rev Oncol Hematol.

[44] Tassinari D, Montanari L, Maltoni M (2007). The palliative prognostic score and survival in patients with advanced solid tumors receiving chemotherapy. Support Care Cancer.

[45] Modonesi C, Scarpi E, Maltoni M (2005). Impact of palliative care unit admission on symptom control evaluated by the Edmonton Symptom Assessment System. J Pain Symptom Manage.

[46] Porzio G, Ricevuto E, Aielli F (2005). The Supportive Care Task Force at the University of L’Aquila: 2-years experience. Support Care Cancer.

[47] Philip J, Smith WB, Craft P, Lickiss N (1998). Concurrent validity of the modified Edmonton Symptom Assessment System with the Rotterdam Symptom Checklist and the Brief Pain Inventory. Support Care Cancer.

[48] Tierney RM, Horton SM, Hannan TJ, Tierney WM (1998). Relationships between symptom relief, quality of life, and satisfaction with hospice care. Palliat Med.

[49] Lam PT, Leung MW, Tse CY (2007). Identifying prognostic factors for survival in advanced cancer patients: a prospective study. Hong Kong Med J.

[50] Paul–Dauphin A, Guillemin F, Virion JM, Briançon S (1999). Bias and precision in visual analogue scales: a randomized controlled trial. Am J Epidemiol.

[51] Bruera E, MacDonald S, Higginson I (1993). Audit methods: the Edmonton Symptom Assessment System. Clinical Audit in Palliative Care.

[52] Regional Palliative Care Program (2001). Guidelines for Using the Edmonton Symptom Assessment System (esas).

[53] Higginson IJ, Sen–Gupta GJ (2000). Place of care in advanced cancer: a qualitative systematic literature review of patient preferences. J Palliat Med.

[54] Homsi J, Walsh D, Rivera N (2006). Symptom evaluation in palliative medicine: patient report vs systematic assessment. Support Care Cancer.

[55] Steinhauser KE, Christakis NA, Clipp EC, McNeilly M, McIntyre L, Tulsky JA (2000). Factors considered important at the end of life by patients, family, physicians, and other care providers. JAMA.

[56] Yennurajalingam S, Zhang T, Bruera E (2007). The impact of the palliative care mobile team on symptom assessment and medication profiles in patients admitted to a comprehensive cancer center. Support Care Cancer.

[57] McCorkle R, Young K (1978). Development of a Symptom Distress Scale. Cancer Nurs.

[58] de Haes JC, van Knippenberg FC, Neijt JP (1990). Measuring psychological and physical distress in cancer patients: structure and application of the Rotterdam Symptom Checklist. Br J Cancer.

[59] Daut RL, Cleeland CS, Flanery RC (1983). Development of the Wisconsin Brief Pain Questionnaire to assess pain in cancer and other diseases. Pain.

[60] National Comprehensive Cancer Network (nccn) (1999). nccn practice guidelines for the management of psychosocial distress. Oncology (Williston Park).

[61] Zigmond AS, Snaith RP (1983). The Hospital Anxiety and Depression Scale. Acta Psychiatr Scand.

[62] Hays RD, Kallich JD, Mapes DL, Coons SJ, Carter WB (1994). Development of the kidney disease quality of life (kdqol) instrument. Qual Life Res.

[63] Steinberg T, Dobson S, Kasymjanova G (2007). Prevalence of emotional distress in newly diagnosed lung cancer patients [abstract]. Support Care Cancer.

[64] Di Dio P, Chasen M, Eades M (2007). Use of the distress thermometer in patients enrolled in a cancer nutrition and rehabilitation program [abstract]. Support Care Cancer.

[65] Higginson I, Higginson I (1993). Audit methods: a community schedule. Clinical Audit in Palliative Care.

[66] Higginson I, Higginson I (1993). Audit methods: validation and in-patient use. Clinical Audit in Palliative Care.

[67] Cohen SR, Mount BM, Strobel MG, Bui F (1995). The McGill Quality of Life Questionnaire: a measure of quality of life appropriate for people with advanced disease. A preliminary study of validity and acceptability. Palliat Med.

[68] Sloan JA, Aaronson N, Cappelleri JC, Fairclough DL, Varricchio C, on behalf of the Clinical Significance Consensus Meeting Group (2002). Assessing the clinical significance of single items relative to summated scores. Mayo Clin Proc.

[69] Nezu AM, Nezu CM, Feurestein M (2007). Psychological distress, depression, and anxiety. Handbook of Cancer Survivorship.

[70] Krohne HW, Davison RJ, Scherer KR, Goldsmith HH (2003). Individual differences in emotional reactions and coping. Handbook of Affective Sciences.

[71] College of Physicians and Surgeons of Ontario (2008). Changing scope of practice. MD Dialogue.

[72] Ceresia P (2007). Medico-Legal Issues Arising from New Health-Care Technologies. Information Sheet 077E.

